# Bladder Cancer Detection Using Electrical Impedance Technique (Tabriz Mark 1)

**DOI:** 10.1155/2012/470101

**Published:** 2012-04-09

**Authors:** Ahmad Keshtkar, Zeinab Salehnia, Asghar Keshtkar, Behrooz Shokouhi

**Affiliations:** ^1^Medical Physics Department, Medical Faculty, Tabriz University of Medical Sciences, Tabriz, Iran; ^2^The Faculty of Engineering and Technology, Imam Khomeini International University (IKIU), Ghazvin, Iran; ^3^The Pathology Department, Medical Faculty, Tabriz University of Medical Sciences, Tabriz, Iran

## Abstract

Bladder cancer is the fourth most common malignant neoplasm in men and the eighth in women. Bladder pathology is usually investigated visually by cystoscopy. In this technique, biopsies are obtained from the suspected area and then, after needed procedure, the diagnostic information can be taken. This is a relatively difficult procedure and is associated with discomfort for the patient and morbidity. Therefore, the electrical impedance spectroscopy (EIS), a minimally invasive screening technique, can be used to separate malignant areas from nonmalignant areas in the urinary bladder. The feasibility of adapting this technique to screen for bladder cancer and abnormalities during cystoscopy has been explored and compared with histopathological evaluation of urinary bladder lesions. Ex vivo studies were carried out in this study by using a total of 30 measured points from malignant and 100 measured points from non-malignant areas of patients bladders in terms of their biopsy reports matching to the electrical impedance measurements. In all measurements, the impedivity of malignant area of bladder tissue was significantly higher than the impedivity of non-malignant area this tissue (*P* < 0.005).

## 1. Introduction

Bladder cancer is an abnormal growth in the urinary bladder. It is primarily a disease of men over 65 and is rarely diagnosed before the age of 40 [1]. Bladder pathology is usually investigated visually by cystoscopy. Erythematous areas of the urothelium are usually observed but these can represent different conditions ranging from simple inflammation to flat carcinoma in situ (CIS). CIS cannot be differentiated visually from other erythematous tissues. Biopsies must be taken from the suspected area to obtain diagnostic information. The selection of biopsy sites depends on simple visual inspection and thus is effectively random and can be negative in up to 90% of the patients [2]. Visual examination of the urinary tract is possible with a cystoscope (a thin, slender tube with a tiny camera attached and a light usually from a fibre optic cable). In this procedure, a cystoscope is placed into the bladder through the urethra and permits the doctor to inspect the inside of the urinary bladder if suspicious areas of growth are seen; a biopsy (removing a small piece of tissue) will be taken for further histopathological examinations. This technique is associated with discomfort for the patient and morbidity; thus, it is important to detect this disease as soon as possible. There are different techniques which currently are available to help with the diagnosis of bladder cancer as follows: diagnostic radiology, excretory urography, urine cytology, CT scan and MRI, ultrasound scans, and cystoscopy. All of the above-mentioned diagnostic techniques can detect the bladder abnormalities after the first stage of the bladder cancer, flat lesions, and carcinoma *in situ*. Although early detection of bladder tumours, prior to muscle invasion, should vastly improve our ability to save both bladders and lives, current methods of detection are neither sufficiently sensitive nor specific [3]. There are several historical studies such as those of Geddes and Baker (1967) which provide useful information about the pathological status of the human body by measuring the bioimpedance of different tissue types [4]. Sugàr, in 1968, carried out an electron microscopic study of the early invasive growth of human skin tumours and laryngeal carcinoma and reported an increase in the extracellular space of these tumours. This may be due to the loss of cellular cohesion [5]. According to Sugar's report, the extracellular spaces in inflammation also increase but this increase is significantly less than in cancerous tissues. In the light of this background, it is possible that electrical impedance spectroscopy may be appropriate for the early detection of flat lesions and assessing bladder pathology. Furthermore, in 1984, White and Gohari investigated epithelial dysplasia by quantifying the volume of the intracellular space in normal and carcinogen-treated hamster cheek-pouch epithelium. They found that there was a significant increase in extracellular space in cancerous tissue [6]. They reported that extra-cellular space in dysplastic tissue (characterized by abnormality of development; alteration in size, shape and organization of cells) was approximately six times the volume of normal tissue. Extra-cellular space increased as dysplasia developed. Thus, the electrical impedance technique can be a novel minimally invasive diagnostic technique to detect bladder cancer and other abnormalities of the human urinary bladder. This is because of the potential of the technique to separate pathologic epithelium from normal epithelium as a result of changes in cell size, cell arrangement, and extra-cellular space. Therefore, the main purpose of this investigation is to study the impedance difference between normal and malignant bladder tissue. 

## 2. Materials and Methods

**Figure 1:**
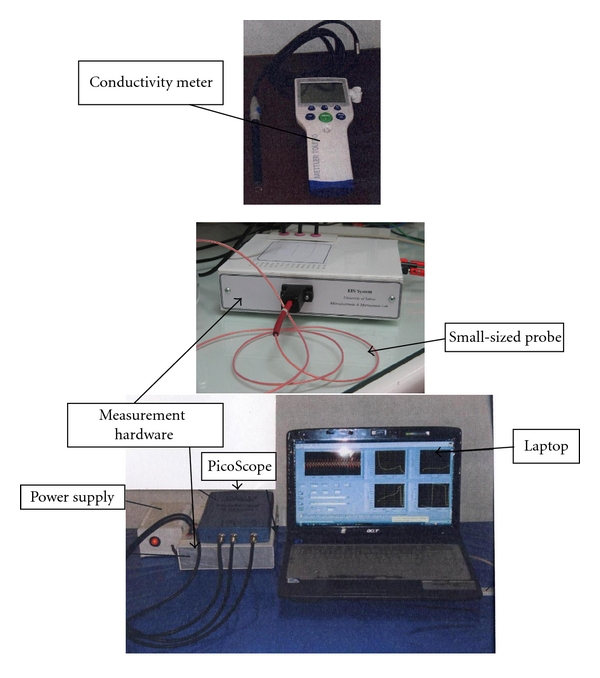
Home-designed impedance measurement system (Tabriz Mark 1).

**Figure 2:**
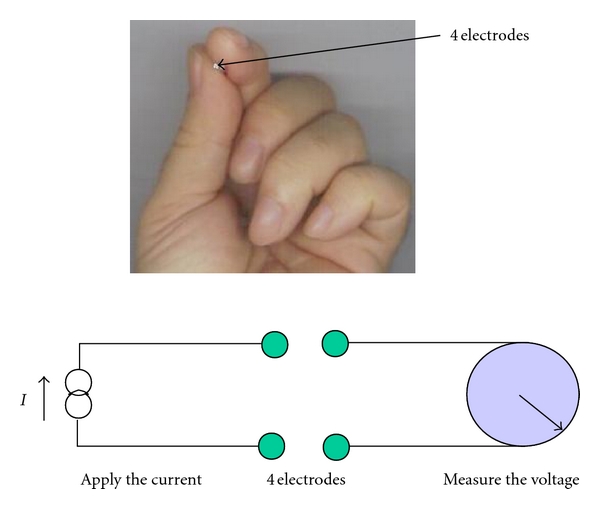
4 Electrodes and their connections to both the current power supply and the voltage measurement system.

**Figure 3:**
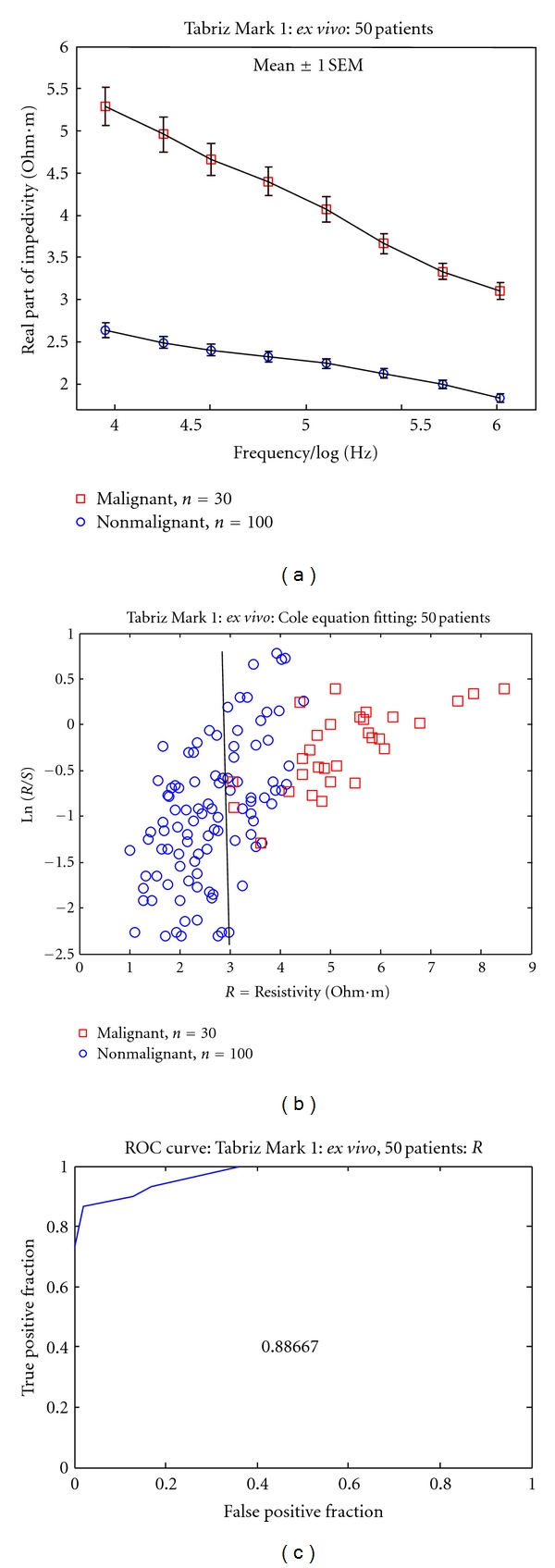
(a) Impedivity of bladder tissue (Ohm·m) against frequency (Hz). (b) Ln*R*/*S* against *R* in Ohm·m. (c) ROC curve for the parameter *R*.

A total of 30 points from malignant and 100 points from non-malignant areas of 50 patients bladders were studied in terms of their biopsy reports matching to the electrical impedance measurements (*ex vivo*). Malignant areas include all of the carcinoma and cancer areas. The electrical impedance of the human urinary bladder was measured using a home-designed impedance measurement system. The living tissue shows a good fitting of impedance against frequency using Cole and Cole equation. It is usual that a wide range of frequency (such as 10 Hz–10 MHz) is used in this type of studies; however, because of some limitations in our PicoScope characteristics we have to limit its range to Figure 3(a). Details can be found in [7–9]. Our system was designed and built in Tabriz University and Tabriz University of Medical Sciences, Tabriz, Iran (Figure 1). We have to mention that we carried out another study to detect the gastric abnormalities using the related electrical impedance by this system [10]. More technical information about our used electrical impedance system is referenced in following papers. In this study, it was introduced a novel low-distortion fully differential current source with very high output impedance using four high-impedance current sources in an H-bridge connection implemented in a standard 180 nm CMOS integrated circuit technology. The small signal simulation result with HSpice shows that an output impedance of 1.66 GΩ can be expected at frequencies up to 1 kHz. The output impedance at 100 MHz is above 109 kΩ for a typical frequency range of up to 10 MHz, and the total harmonic distortion remains about 1%. By properly rationing the size of the current source transistors, a desired output impedance characteristic can be achieved [11]. This system combines with a laptop computer and picoscope (PC Oscilloscope) using measurement hardware (Figure 1). The constant current, 10 microampere, is passing through two electrodes of a small-sized probe. The resulting potential was measured using other two electrodes to obtain the electrical impedance of the bladder tissue. To explain this probe in detail, the electrodes are constructed of four gold wire electrodes, 0.5 mm in diameter, spaced equally on a 1.6 mm diameter circle (total diameter of the probe was only 2 mm). In the measuring procedure using this probe, it did not matter which pair of electrodes, current injection electrodes, or potential measurement electrodes, was used. Detailed information about this probe can be found in Keshtkar (2006) [12]. The most common form of measuring tissue impedance is the tetrapolar or the four-electrode technique. We can measure the transfer impedance (the ratio of the measured voltage to the applied current) of the living tissue with this technique [13]. Brown et al. used this technique in different studies [14, 15]. It was used because it is designed to minimize the effect of electrode impedance on the measured data (see Figure 2): In the impedance data collection procedure, the probe was regularly calibrated using the known conductivity of saline solutions before any measurement procedure to have the tissue impedance readings in terms of the impedivity in Ωm. All constructed probes were calibrated using known conductivity saline solutions; different saline solutions were prepared using a conductivity meter. These conductivities cover the full range of interest for bladder tissue conductivity. Then, the respective readings of every solution were taken using the electrical impedance measurement system and then stored in a portable computer (laptop) for further analysis. Therefore, the principle of the calibration procedure was to relate the measured transfer impedance to a known uniform conductivity, by means of a calibration factor or probe, constant for each measurement frequency. These factors applied to the computer readings using Matlab-based programme to obtain the actual electrical impedance measured data in Ohm·m. In impedance measuring procedure, after obtaining each impedance reading a biopsy was taken from the measurement area and then fixed separately in formalin solution for histopathological examination (to compare the measured data with the pathological results). However, every measurement consists of the mean value of 5 spectral measurements in order to check the reproducibility of the measurements. Of course, there are poor results for small-sized probe in terms of reproducibility of the measurements and it needs further studies [16]. It must be mentioned that, in order to analyze data, a simple tissue equivalent model, the Cole equation model, was used in this study. This model considers the tissue as a combination of two resistors and a capacitance: one resistor (*R*), the extracellular space is in parallel with a series of a membrane capacitance (*C*), and another resistor (*S*), the intracellular space. The important matter that must be considered is that the electrical impedance drops with an increase in frequency [13]. Furthermore, to evaluate the significance of separating measured impedance spectrum, a nonparametric statistical test, the Kruskal-Wallis test, was applied to these data (the impedance data were not distributed normally due to the boxplots resulting from the measured data) [17]. The measured impedance data were recorded in a laptop computer for further data analysis. 

## 3. Results

The probe constant or the calibration factor (conversion factor) resulted from the probe calibration procedure to change the measurement system readings to the impedivity in Ωm. In fact, these factors were calculated and averaged for each frequency using simple M-file written in Matlab program. By considering the probe calibration factor, the amounts of electrical impedance, impedivity, or resistivity were calculated for benign and tumoral areas of the bladder tissue (with considering their pathological reports). Then, the relationship between the tissue impedance and frequency for these areas is shown in Figure 3(a). According to the calculation of respective data for the measurements using this system, the resistivity of malignant group was higher than that of the non-malignant group (*P* < 0.005).  Figure 3(b) demonstrated that Ln*R*/*S* against *R* in Ohm·m and showed rectangular and circle points related to malignant and non-malignant areas, respectively. This figure shows that a high percentage of malignant points usually are in the right-hand side of non-malignant points. ROC curves is the receiver operating characteristic curve and plotted in this study (Figure 3(c)); this curve shows a good separation between malignant and non-malignant tissues in terms of their extracellular space (*R*). The under curve area in this plot is 0.886.

## 4. Discussion

In this study, the impedivity of malignant area of the bladder tissue is significantly higher than that of the non-malignant areas (Figure 3(a)). It is known that at lower frequencies the current flows mainly through the extra-cellular space, whereas at higher frequencies the current flows both through the extra-cellular space as well as through the intracellular space. Also, in this figure, the tumor tissue (has the lack of tight junction) has electrical impedance a less than the impedance of normal tissue, and therefore, this is probably because of the lack of tight junction in their tissue structure. This might be associated with the fact that tumor cells have higher water content than normal cells [18–21]. Furthermore, according to the Cole equation model, the malignant and non-malignant points are placed (approximately) on the right-hand side and left-hand side of Figure 3(b), respectively. Thus a high percentage of malignant points usually are in the right-hand side of non-malignant points. Finally, there is only a grouped data separation and not a perfect categorization of the individual measurements. Moreover, to distinguish the individual data, which are not placed in its groups, one can consider the receiver operating characteristic (ROC) curves for the parameters.

## 5. Conclusion

The electrical impedance data of the bladder epithelium were measured and compared with histopathological reports of the biopsies, which had been taken from the measurement area. The final results show that the resistivity of the malignant group was higher than that of the non-malignant groups (*P* < 0.005). The Cole equation fitting procedure was used to generate a scatter plot of the malignant and non-malignant points. A high percentage of malignant points usually are in the right-hand side of non-malignant points using scattering plots that resulted from the Cole and Cole equation. Therefore, electrical impedance spectroscopy can be a useful technique to characterize the bladder tissue and this can be a complementary method for endoscopy, biopsy, and histopathological evaluation of the bladder abnormalities.
